# miR-455-5p enhances 5-fluorouracil sensitivity in colorectal cancer cells by targeting PIK3R1 and DEPDC1

**DOI:** 10.1515/med-2022-0474

**Published:** 2022-04-28

**Authors:** Tingting Lou, Luqing Zhang, Zongshan Jin, Chundi Miao, Jinqiu Wang, Kongliang Ke

**Affiliations:** Department of General Surgery, Ningbo Hangzhou Bay Hospital, Hangzhou Bay New District, Ningbo 315300, Zhejiang Province, China; Department of Anesthesiology, Ningbo Hangzhou Bay Hospital, Ningbo, Zhejiang Province, China; Department of Breast and Thyroid surgery, Ningbo First Hospital, Ningbo, Zhejiang Province, China; Department of General Surgery, Ningbo Hangzhou Bay Hospital, No. 1155, Binhai No. 2 Road, Hangzhou Bay New District, Ningbo 315300, Zhejiang Province, China

**Keywords:** miR-455-5p, 5-fluorouracil sensitivity, colorectal cancer, PIK3R1, DEPDC1

## Abstract

Our previous study has demonstrated that miR-455-5p was a tumor suppressor in colorectal cancer (CRC). This study aimed to investigate the role of miR-455-5p in 5-fluorouracil (5-Fu) in CRC. The expression of miR-455-5p, PIK3R1, and DEPDC1 was analyzed in HT-29 cells after treatment with different concentrations (0, 0.5, 2.5, and 12.5 μM) of 5-Fu. The effects of miR-455-5p on cell proliferation and apoptosis were analyzed by CCK-8 and flow cytometry. PIK3R1 and DEPDC1 were overexpressed to measure the mechanism of miR-455-5p on 5-Fu sensitivity. And the direct binding between miR-455-5p and DEPDC1 was detected by a dual-luciferase reporter assay. We found that miR-455-5p decreased, while PIK3R1 and DEPDC1 increased after 5-Fu treatment. miR-455-5p mimic significantly suppressed cell viability and elevated cell apoptosis in 5-Fu-treated HT-29 cells, whereas miR-455-5p inhibitor showed the opposite effects. Overexpression of PIK3R1 and DEPDC1 could attenuate the effects of miR-455-5p mimic on the viability and apoptosis of 5-Fu-treated cells. miR-455-5p could directly bind to DEPDC1 in HT-29 cells. In conclusion, miR-455-5p enhanced 5-Fu sensitivity by targeting PIK3R1 and DEPDC1 in CRC. This study provides a novel role of miR-455-5p in CRC and restoring miR-455-5p might be a therapeutic strategy to enhance chemosensitivity to 5-Fu.

## 1 Introduction

Colorectal cancer (CRC) is the third most commonly diagnosed cancer and the second leading cause of cancer death in the world, with approximately 1.93 million new cases and 935,000 deaths in 2020 [1]. Currently, the treatment methods of CRC include surgery, chemotherapy, and radiotherapy [2]. However, surgery is the only curative treatment, especially for patients with early stages [3]. About 25% of CRC patients exhibit metastasis at diagnosis, when further treatments with adjuvant chemotherapy and/or radiotherapy are needed [2,4]. Because of the emergence of resistance, patients become tolerant to these adjuvant treatments, resulting in tumor recurrence [3]. It has been reported that the 5-year survival rate of patients with metastatic CRC is low, remaining only about 14% [4]. 5-Fluorouracil (5-Fu) is a most commonly used chemotherapy drug for CRC patients [3]. Hence, it is important to explore a method to improve the 5-Fu sensitivity in CRC cells.

MicroRNAs (miRNAs) are endogenously expressed small noncoding RNA, with approximately 21–25 nucleotides in length. They have been demonstrated to regulate cancer progression via negatively modulating gene expression by inducing target mRNA degradation and/or blocking translation [5]. Aberrantly expressed miRNAs are related to tumor proliferation and metastasis, and act as tumor promotor or suppressor. In recent years, more and more researchers have focused on their role in drug resistance. For example, Huynh et al. [6] found that miR-221 contributed to acquired lapatinib resistance via negatively regulating p27 expression in HER2-positive breast cancer. miR-138-1-3p enhanced sorafenib sensitivity in hepatocellular carcinoma by regulating PAK5-mediated β-catenin/ABCB1 signaling pathway [7]. miR-375-3p, which was downregulated in human CRC cell lines and tissues, has been reported to promote the sensitivity of CRC cells to 5-Fu by targeting thymidylate synthase [8].

In the last decade, miR-455-5p has been validated to be abnormally expressed and plays a critical role in multiple cancers. On one hand, miR-455-5p acted as a tumor promotor. For instance, higher miR-455-5p expression was found in oral squamous cancer (OSCC) tissues than that in the normal samples, and its expression level was related to the nodal status, stage, and overall survival in OSCC patients. miR-455-5p promoted the proliferation of OSCC cells via targeting ubiquitin-conjugating enzyme E2B [9]. miR‑455‑5p was also upregulated in breast cancer, and the increase of miR-455-5p was associated with patients’ poor survival rate. miR‑455‑5p could promote the invasion and migration of breast cancer cells via targeting programmed cell death 4 [10]. On the other hand, miR-455-5p could also function as a tumor inhibitor. For example, decreased miR-455-5p expression predicted a poor prognosis of prostate cancer, and its upregulation significantly inhibited tumor growth and triggered apoptosis via negatively mediating C–C motif chemokine receptor 5 [11]. Overexpression of mR-455-5p suppressed cholangiocarcinoma growth and metastasis via targeting protein phosphatase 1 regulatory subunit 12A, leading to the inactivation of mitogen-activated protein kinases and PI3K/AKT pathways [12]. Liu et al. [13] found that miR-455-5p expression was decreased in gastric cancer. And overexpression of miR-455-5p could suppress gastric cancer proliferation and invasion, but facilitate cell apoptosis. Our previous study has found that miR-455-5p expression was downregulated in CRC tissues when compared to adjacent normal tissues. And miR-455-5p could suppress cell proliferation and migration, whereas promote cell apoptosis in CRC cells [14]. However, the effect of miR-455-5p on 5-Fu sensitivity in CRC cells remains unclear.

In this study, we first analyzed the influence of 5-Fu treatment on miR-455-5p expression in CRC cells. Subsequently, miR-455-5p mimic and inhibitor were used to determine the role of miR-455-5p in 5-Fu sensitivity. Additionally, the underlying mechanism was further explored.

## 2 Materials and methods

### 2.1 Cell culture and treatment

CRC cell line HT-29 was provided by Shanghai Nuobai Pharmaceutical Co., Ltd (Shanghai, China) and cultured in dulbecco’s modified eagle’s medium (DMEM) as our previous study [14]. HT-29 cells in logarithmic phase were digested by trypsin and planted into indicated cell culture plates. After culturing for 24 h, the cells were treated with different concentrations (0, 0.5, 2.5, and 12.5 μM) of 5-Fu for 6, 24, 48, and 72 h.

### 2.2 Cell Counting Kit-8 (CCK-8) assay for cell viability

A total of 2  ×  10^4^ HT-29 cells transfected with or without indicated vector for 24 h were seeded into 96-well plates and cultured in DMEM containing 10% fetal bovine serum for 24 h. Subsequently, cell culture medium was changed to that contains designated concentrations of 5-Fu. After maintaining for indicated times, 10 μL of CCK-8 solution (CK04-05, DOJINDO, Japan) was added to each well for 2 h. Then, absorbance was measured using a microplate reader (RT-2100C, Rayto, USA) at a wavelength of 450 nm.

### 2.3 Cell apoptosis analysis

The apoptosis of HT-29 cells was detected by flow cytometry using an Annexin V-PE/7-AAD apoptosis assay kit (Nanjing KeyGen Biotech Co., Ltd., Nanjing, China). Briefly, HT-29 cells were planted into 6-well plates. After culturing for 24 h, cells were incubated with 2.5 μM 5-Fu for 48 h. Cells were collected and washed twice with PBS. Then, cells were incubated 15 min in the dark at room temperature with 5 μL of 7-AAD solution, which was dissolved in 50 μL of Binding Buffer. After adding 450 μl of Binding Buffer, cells were maintained with Annexin V-PE (1 μL) for 15 min in the dark. Finally, cells were measured by a BD FACS Calibur Flow Cytometry System (BD Biosciences, Franklin Lakes, NJ, USA). Annexin V-PE-positive cells were regarded as apoptotic cells.

### 2.4 Plasmid construction and transfection

Full-length complementary DNAs (cDNAs) of phosphoinositide-3-kinase regulatory subunit 1 (PIK3R1) and DEP domain containing 1 (DEPDC1) were amplified from HT-29 cells, and inserted into pcDNA3.1 plasmid to construct PIK3R1 and DEPDC1 overexpressing plasmids. pcDNA empty vector was used as a negative control. miR-455-5p mimic, inhibitor, and their controls were provided by Shanghai Nuobai Pharmaceutical Co., Ltd. Constructed recombinant plasmids, miR-455-5p mimic, inhibitor, and corresponding controls were transfected into HT-29 cells using Lipofectamine 2000 reagent (Invitrogen, Carlsbad, CA, USA) based on the manufacturer’s instructions.

### 2.5 Bioinformatics analysis

The target of miR-455-5p was predicated by Starbase v3.0 software (http://www.sysu.edu.cn/). GSE38061 was downloaded from the Gene Expression Omnibus (GEO) database (https://www.ncbi.nlm.nih.gov/geo/), and differentially expressed genes in 5-Fu-treated HCT116 cells were analyzed by GEO2R with thresholds of |log2 FC (fold-change)| >1 and *P* < 0.01.

### 2.6 Dual-luciferase reporter assay

The potential binding site of DEPDC1 (DEPDC1-WT) and its mutant sequence (DEPDC1-MUT) were cloned into the pmirGLO dual-luciferase reporter vector. Then, these plasmids were transfected into HT-29 cells with miR-455-5p mimic or mimic control. The luciferase activity of cells was measured by a dual-luciferase assay system (Promega, Madison, WI, USA) after 48 h. The firefly luciferase activity was normalized to that of Renilla luciferase internal control.

### 2.7 Real-time PCR

Total RNA from HT-29 cells was extracted by Trizol Reagent (Invitrogen). SuperScriptIII Reverse Transcriptase (Invitrogen) was used to synthesize cDNA. The real-time PCR reaction systems were performed on CFX96TM Real-Time System (Bio-Rad, Hercules, CA, USA) using Platinum Taq DNA Polymerase (Invitrogen). Gene relative expression was processed using the 2^−ΔΔCt^ method. The relative amounts of miR-455-5p were normalized to miR-16 [15], and β-actin was used as an endogenous control of PIK3R1 and DEPDC1. Primer sequences are provided in Table 1.

**Table 1:** Primer sequences

Gene	Primer sequences
miR-455-5p	RT: 5′-CTCAACTGGTGTCGTGGAGTCGGCAATTCAGTTGAGCGATGTAG-3′
	F: 5′-ACACTCCAGCTGGGTATGTGCCTTTGGACT-3′
	R: 5′-TGGTGTCGTGGAGTCG-3′
miR-16	RT: 5′-CTCAACTGGTGTCGTGGAGTCGGCAATTCAGTTGAGCGCCAATA-3′
	F: 5′-ACACTCCAGCTGGGTAGCAGCACGTAAATA-3′
	R: 5′-TGGTGTCGTGGAGTCG-3′
PIK3R1	F: 5′-GGTGAAGCTCGTGTGTGGA-3′
	R: 5′-CAGCAGAGGACAAAGGACCA-3′
DEPDC1	F: 5′-GCTACAAGTAAAGAGGGGATGG-3′
	R: 5′-GGACAGAAAGGTAAGTCAGTGGG-3′
β-Actin	F: 5′-ACACCCCAGCCATGTACGT-3′
	R: 5′-ATGGGCACAGTGTGGGTGA-3′

### 2.8 Western blot

Total proteins from HT-29 cells were isolated using RIPA lysis buffer. The protein concentration was tested by a bicinchoninic acid protein assay kit (Thermo Fisher Scientific, Rockford, USA). Then, the western blot assay was carried out as described earlier [14]. Both PIK3R1 (1:1000) and DEPDC1 (1:800) primary antibodies were from Abcam (Cambridge, MA, USA).

### 2.9 Statistical analysis

All experiments were repeated three times and data were presented as mean ± standard deviation. The statistical analysis was performed with Student’s *t* test and one-way analysis of variance using Graphpad Prism 8 software. *P* < 0.05 was considered as statistically significant.


**Ethical approval:** The conducted research is not related to either human or animals use.

## 3 Results

### 3.1 miR-455-5p expression decreased in 5-Fu-treated CRC cells in a dose- and time-dependent manner

**Figure 1:**
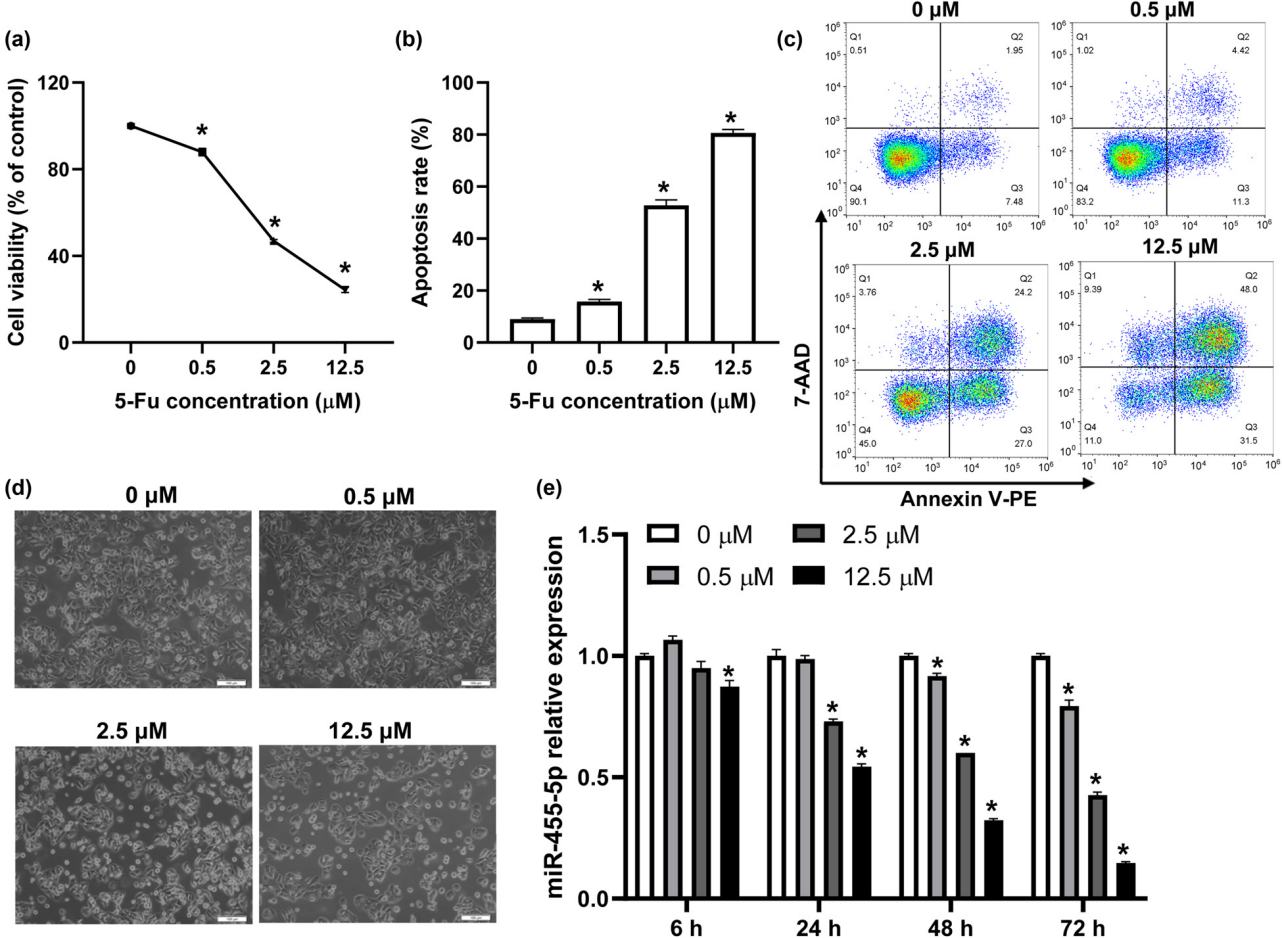
miR-455-5p expression decreased in 5-Fu-treated CRC cells. (a) HT-29 cells were treated with different concentrations of 5-Fu for 48 h, CCK-8 was used to assay cell viability. (b and c) Flow cytometry was used to detect the apoptosis of HT-29 cells under different treatments. (d) Cell morphology was observed with a microscope. (e) The expression of miR-455-5p was analyzed by real-time PCR in HT-29 cells after treatment with 5-Fu (0, 0.5, 2.5, and 12.5 μM) for 6, 24, 48, and 72 h. ^*^ *P* < 0.05 vs the control (0 μM) group.

HT-29 cells were treated with different concentrations of 5-Fu (0, 0.5, 2.5, and 12.5 μM), cell viability was significantly suppressed, and cell apoptosis was elevated by 5-Fu in a dose-dependent manner (Figure 1a–c). Also, cell death was observed under a microscope and the results revealed that 5-Fu damaged the morphological integrity of cells, with plenty of cellular debris in cells treated with 5-Fu at concentrations of 2.5 and 12.5 μM (Figure 1d). These results indicated that 5-Fu remarkably inhibited CRC cell growth. Furthermore, we investigated the influence of 5-Fu on miR-455-5p expression by using real-time PCR. The data revealed that miR-455-5p expression was reduced by 5-Fu in a dose- and time-dependent manner (Figure 1e).

### 3.2 miR-455-5p sensitizes CRC cells to 5-Fu

**Figure 2:**
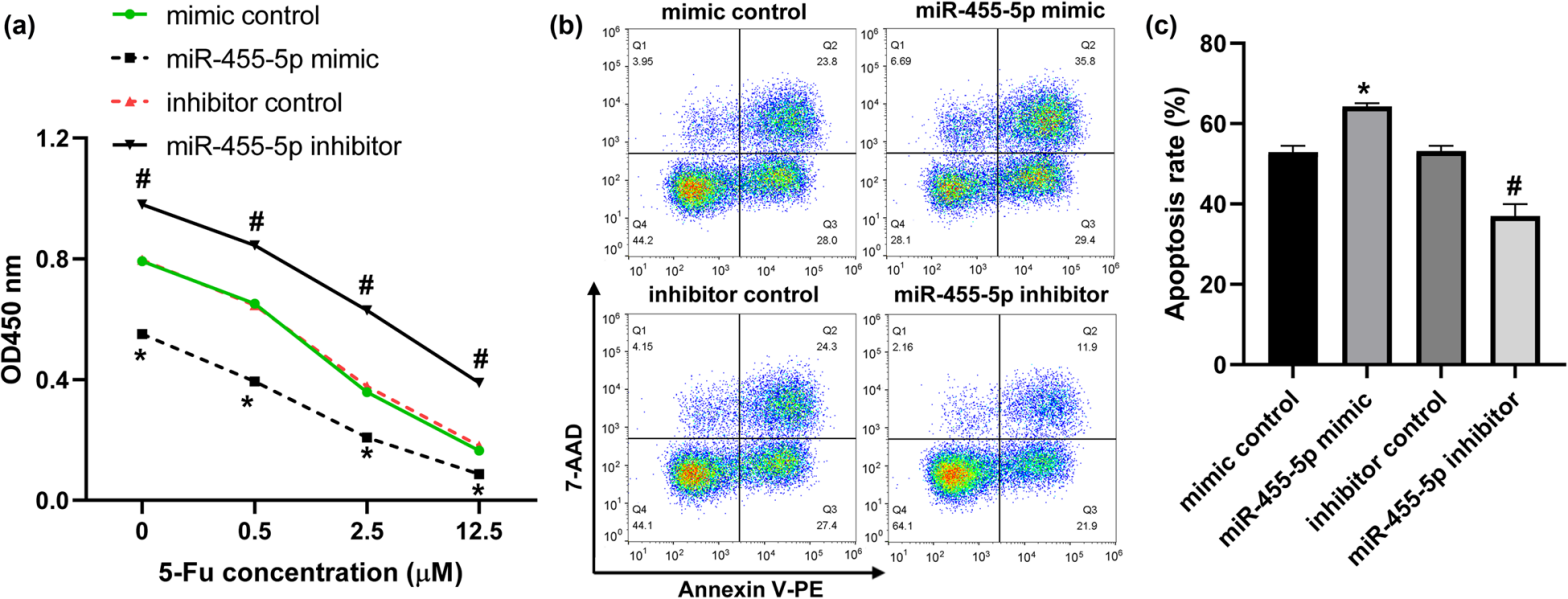
miR-455-5p sensitizes CRC cells to 5-Fu. (a) HT-29 cells were transfected with miR-455-5p mimic, inhibitor, and their corresponding control for 48 h, cell viability was analyzed by CCK-8. (b and c) The apoptosis of HT-29 cells was detected by flow cytometry. ^*^ *P* < 0.05 vs the mimic control, ^#^ *P* < 0.05 vs the inhibitor control.

Next, we further explored the effect of miR-455-5p on 5-Fu sensitivity in CRC cells. As shown in Figure 2a, miR-455-5p mimic notably decreased, whereas miR-455-5p inhibitor increased the viability of HT-29 cells treated with or without different concentrations of 5-Fu (Figure 2a). Moreover, miR-455-5p mimic promoted the apoptosis of CRC cells under 5-Fu treatment (Figure 2b and c). And compared with the inhibitor control, miR-455-5p inhibitor suppressed the apoptosis of CRC cells treated with 5-Fu (Figure 2b and c). These results indicated that miR-455-5p promoted 5-Fu sensitivity in CRC cells.

### 3.3 PIK3R1 was a target of miR-455-5p in 5-Fu-treated CRC cells

**Figure 3:**
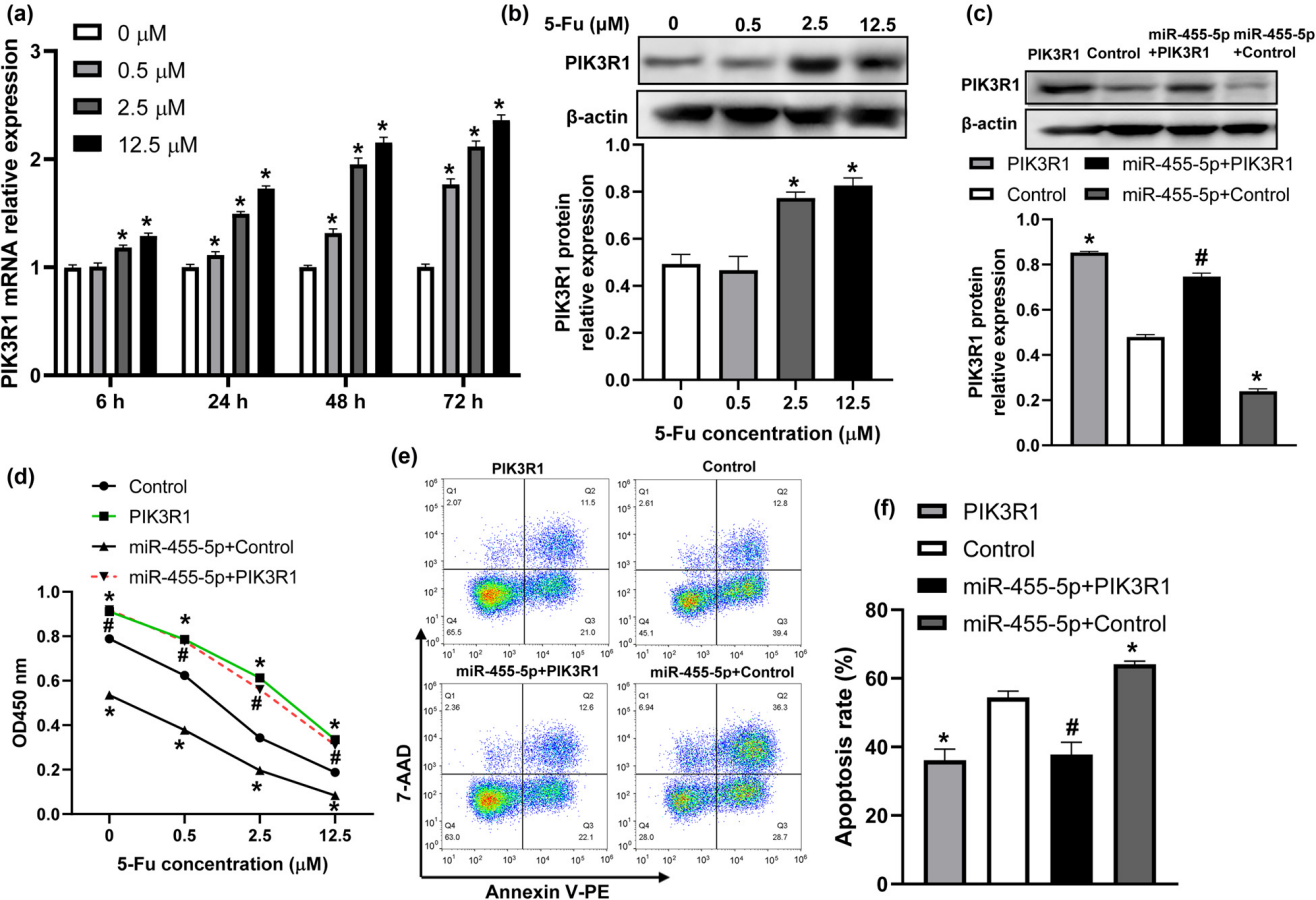
PIK3R1 was a target of miR-455-5p in 5-Fu-treated CRC cells. (a) PIK3R1 mRNA expression was detected in HT-29 cells after treatment with different concentrations of 5-Fu for 6, 24, 48, and 72 h. (b) PIK3R1 protein level was analyzed in HT-29 cells treated with 5-Fu for 48 h. (c) HT-29 cells were transfected with PIK3R1 overexpressing plasmid or pcDNA3.1 control plasmid, or co-transfected with miR-455-5p mimic and PIK3R1 overexpressing plasmid or pcDNA3.1 control plasmid for 24 h, and then exposed to 2.5 μM 5-Fu for 48 h. PIK3R1 protein level was measured by western blot. (d) Cell viability was determined by CCK-8. (e and f) Flow cytometry was used to assay the apoptosis of HT-29 cells under different treatments. ^*^ *P* < 0.05 vs the control group, ^#^ *P* < 0.05 vs the miR-455-5p + control group.

Our previous study showed that PIK3R1 was a target of miR-455-5p in CRC cells [14]. And silencing of PIK3R1 in CRC cells enhanced 5-Fu-induced apoptosis [16]. Hence, we analyzed whether miR-455-5p regulated 5-Fu sensitivity in CRC cells via targeting PIK3R1. As shown in Figure 3a and b, the mRNA and protein expression of PIK3R1 increased in CRC cells treated with 5-Fu. miR-455-5p suppressed PIK3R1 protein expression in CRC cells treated with 5-Fu (Figure 3c). Overexpression of PIK3R1 significantly elevated the viability and reduced the apoptosis in HT29 cells (Figure 3d–f). Moreover, upregulation of PIK3R1 attenuated the effects of miR-455-5p mimic affected cell viability and apoptosis (Figure 3d–f). These results indicated that miR-455-5p elevated 5-Fu sensitivity in CRC cells partly via targeting PIK3R1.

### 3.4 DEPDC1 is another target of miR-455-5p in CRC cells

**Figure 4:**
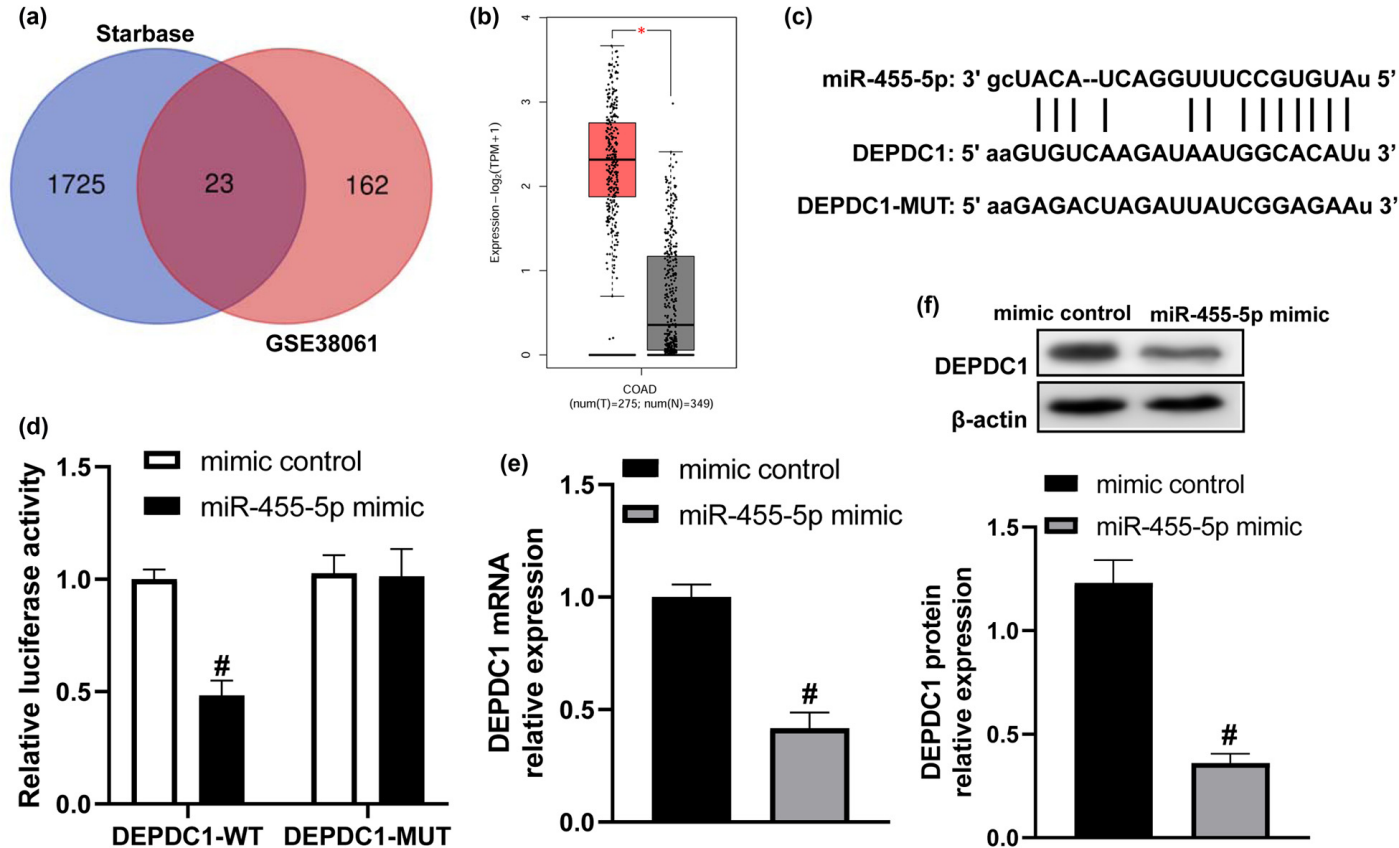
DEPDC1 is a target of miR-455-5p in CRC cells. (a) Venn diagram of specific genes between potential miR-455-5p target genes from Starbase and differentially expressed genes from the GEO dataset GSE38061. (b) GEPIA2 software assay for DEPDC1 expression in colon adenocarcinoma (COAD) tissues and normal samples. (c) The binding sites between miR-455-5p and DEPDC1. (d) HT-29 cells were co-transfected with DEPDC1-WT/DEPDC1-MUT luciferase reporter vectors and miR-455-5p mimic/mimic control for 48 h, and the luciferase activity was detected. (e and f) miR-455-5p mimic significantly decreased the DEPDC1 mRNA and protein levels in HT-29 cells. ^*^ *P* < 0.05 vs normal tissues, ^#^ *P* < 0.05 vs the mimic control group.

Studies have demonstrated that one miRNA can regulate multiple genes [17,18]. Hence, we hypothesized that miR-455-5p might target another gene besides PIK3R1. We analyzed the differentially expressed genes in HCT116 cells after 5-Fu treatment in GSE38061 dataset and the target genes of miR-455-5p by Starbase software, and found that 23 candidate genes (PLK1, TRIM2, PLCL2, SECISBP2, BTBD2, DUSP1, ZNF561, SOCS4, SLC7A11, ZMAT3, PRDM1, DEPDC1, SLC7A2, TP53INP1, H6PD, PMAIP1, TNFRSF10B, TGFA, SLC39A10, F3, ZNF804A, TNFRSF10D, and HIST2H2BE) coexisted in these two datasets (Figure 4a). Among these genes, DEPDC1 was selected. Because DEPDC1 expression increased in CRC tumor tissues (Figure 4b) and previous study has demonstrated that high expression of DEPDC1 was related to the poor tumor node metastasis (TNM) stage and recurrence [19]. Also, it could promote cell proliferation, migration, invasion, and epithelial–mesenchymal transition of CRC [20]. It was shown to decrease the sensitivity to chemotherapy in hepatocellular carcinoma [21]. The binding sites between miR-455-5p and DEPDC1 are revealed in Figure 4c. Dual-luciferase assay indicated that miR-455-5p mimic only inhibited the luciferase activity of cells transfected with DEPDC1-WT plasmid (Figure 4d). Moreover, from the real-time PCR and western blot assays, the results showed that miR-455-5p mimic markedly reduced DEPDC1 expression both at transcription and translation levels (Figure 4e and f). These data suggested that DEPDC1 was a target of miR-455-5p in CRC cells.

### 3.5 miR-455-5p enhanced 5-Fu sensitivity of CRC cells via modulating DEPDC1

**Figure 5:**
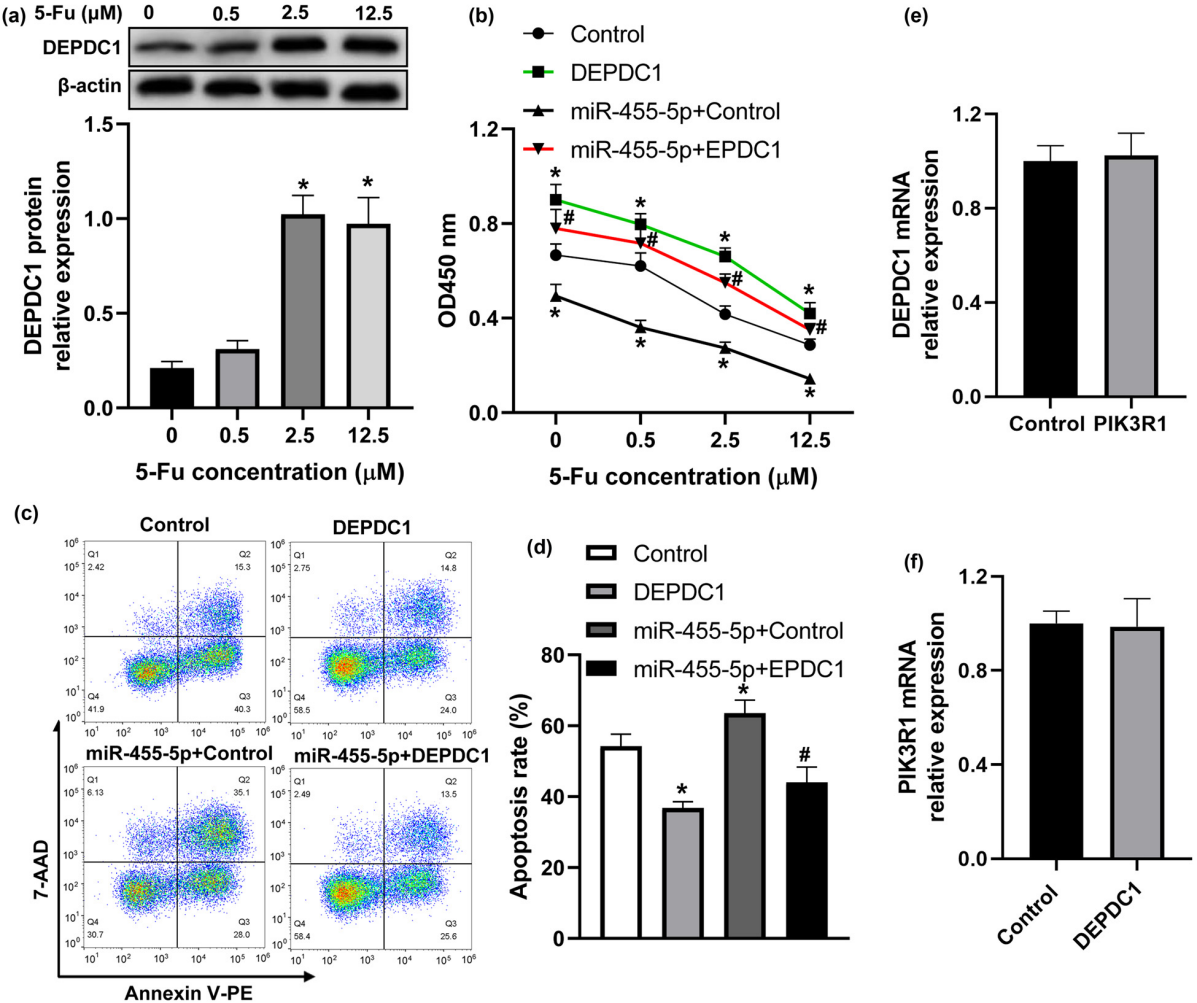
miR-455-5p enhanced 5-Fu sensitivity of CRC cells via modulating DEPDC1. (a) DEPDC1 protein expression was analyzed by western blot in HT-29 cells after treatment with different concentrations of 5-Fu (0, 0.5, 2.5, and 12.5 μM) for 48 h. (b) Cell viability was detected by CCK-8. (c and d) The apoptosis of HT-29 cells was measured by flow cytometry. (e) DEPDC1 expression was analyzed by real-time PCR in HT-29 cells transfected with PIK3R1 overexpressing plasmid or pcDNA3.1 control plasmid. (f) PIK3R1 mRNA expression in HT-29 cells transfected with DEPDC1 overexpressing plasmid or pcDNA3.1 control plasmid. ^*^ *P* < 0.05 vs the control group, ^#^ *P* < 0.05 vs the miR-455-5p + control group.

Subsequently, we further analyzed whether miR-455-5p regulated 5-Fu sensitivity via DEPDC1. DEPDC1 expression was elevated in 5-Fu-treated cells (Figure 5a). Upregulation of DEPDC1 significantly promoted the viability, whereas it inhibited the apoptosis of CRC cells (Figure 5b–d). Moreover, DEPDC1 reversed the effects of miR-455-5p on the viability and apoptosis of CRC cells (Figure 5b–d). Taken together, these data indicated that miR-455-5p elevated 5-Fu sensitivity of CRC cells via targeting DEPDC1. Additionally, we examined whether PIK3R1 and DEPDC1 had interaction with each other, and the results showed that overexpression of PIK3R1 could not affect DEPDC1 expression (Figure 5e). At the same time, PIK3R1 expression was not altered by DEPDC1 in HT-29 cells (Figure 5f). These results indicated that these two candidates have no interaction with each other.

## 4 Discussion

In this study, we found that miR-455-5p decreased after 5-Fu treatment in CRC cells. miR-455-5p overexpression notably promoted 5-Fu sensitivity, whereas its inhibitor decreased 5-Fu sensitivity in CRC cells. The mechanism investigation suggested that miR-455-5p participated 5-Fu resistance via targeting PIK3R1 and DEPDC1.

Accumulating evidence has demonstrated that miRNAs play a critical role in drug resistance [6,8]. In the past, we found that miR-455-5p suppressed the proliferation and migration, whereas it promoted the apoptosis of CRC cells [14]. The characteristics of tumor cells, including proliferation, migration, and apoptosis, are closely associated with their sensitivity to chemotherapy [8,22]. Chen et al. [23] have revealed that miR-455-5p promoted cisplatin sensitivity in bladder cancer. 5-Fu resistance is the main cause of failure in the chemotherapy of CRC [3]. Hence, in the current study, we analyzed the effect of miR-455-5p on 5-Fu sensitivity in CRC cells. 5-Fu exposure significantly decreased CRC cell proliferation and induced apoptosis. Furthermore, 5-Fu markedly decreased miR-455-5p expression in a dose- and time-dependent manner. Our results also found that miR-455-5p mimic significantly suppressed the proliferation, but elevated the apoptosis in 5-Fu-treated CRC cells. Contrarily, miR-455-5p inhibitor showed the opposite effects on the proliferation and apoptosis in CRC cells. These results validated that miR-455-5p could enhance the sensitivity of CRC cells to 5-Fu by inhibiting proliferation and promoting apoptosis.

miRNA plays a role in multiple biological process via regulating its downstream target [24]. And we further analyzed the target of miR-455-5p in 5-Fu-treated CRC cells. Our previous study showed that PIK3R1 was a target of miR-455-5p in CRC cells [14]. PIK3R1 encodes the regulatory subunit of PI3K (p85α), a regulator of PI3K/Akt signaling, that is involved in tumorigenesis [25]. Studies have shown that PIK3R1 was responsible for chemotherapy resistance in many cancers [26,27]. And analyzing the public GEO database (GSE122860), we found that PIK3R1 expression was elevated in 5-Fu-treated HCT116 CRC cells (data were not shown). Consistently, this study also revealed that PIK3R1 expression was upregulated after 5-Fu treatment. Upregulation of PIK3R1 led to an increase of cell viability, and a decrease of cell apoptosis in 5-Fu-treated CRC cells. This confirmed that PIK3R1 could reduce the sensitivity of CRC cells to 5-Fu. Moreover, our results showed that PIK3R1 overexpression significantly attenuated the effects of miR-455-5p mimic on 5-Fu-affected cell viability and apoptosis in CRC cells. In general, these results indicated that miR-455-5p enhanced 5-Fu sensitivity in CRC cells via negatively regulating PIK3R1.

DEPDC1 is a newly identified tumor-promotor gene that is abnormally expressed in many cancers and contributes to tumorigenesis, such as breast cancer [28], gastric cancer [29], and lung adenocarcinoma [30]. Similarly, DEPDC1 expression was elevated in CRC. Studies have confirmed that high expression of DEPDC1 was related to the poor TNM stage and recurrence [19], and DEPDC1 could promote proliferation, invasion, and epithelial–mesenchymal transition in CRC via elevating the expression of suppressor of zest 12 [20]. Importantly, increased DEPDC1 has been found to suppress the sensitivity to chemotherapy in hepatocellular carcinoma [21]. By bioinformatics analysis, we found that DEPDC1 coexisted in the potential targets of miR-455-5p and the differentially expressed genes in HCT116 cells after 5-Fu treatment in the GSE38061 database. Hence, we further investigated whether miR-455-5p regulated 5-Fu sensitivity in CRC via DEPDC1. Through dual-luciferase assay and expression level examination, we verified that DEPDC1 was a target of miR-455-5p in CRC. As expected, DEPDC1 expression was elevated in CRC cells after 5-Fu treatment. And upregulation of DEPDC1 attenuated the effects of miR-455-5p on the viability and apoptosis of 5-Fu-treated cells. These results suggested that miR-455-5p elevated 5-Fu sensitivity of CRC cells via targeting DEPDC1. Noteworthy, we also found that PIK3R1 and DEPDC1 could not affect the expression of each other, which indicated that these two candidates were both important for miR-455-5p functioning.

At first sight, it seems somewhat contradictory that 5-Fu treatment increased the PIK3R1 and DEPDC1 expression, and overexpression of PIK3R1 and DEPDC1 could promote cell viability and inhibit the apoptosis of CRC cells. The regulation of cell proliferation and apoptosis is a dynamic equilibrium state, which is mediated by multiple oncogenes and tumor suppressors in cells. 5-Fu treatment increased the expression of PIK3R1 and DEPDC1, which was the anti-apoptotic response of cells. Hence, our conclusions that 5-Fu markedly decreased miR-455-5p expression, and miR-455-5p could enhance 5-Fu sensitivity in CRC cells via negatively regulating PIK3R1 and DEPC1 were not contradictory.

In conclusion, this study elucidated a novel role of miR-455-5p in regulating 5-Fu sensitivity in CRC. It elevated the sensitivity of CRC cells to 5-Fu via targeting PIK3R1 and DEPDC1. However, this study has some limitations, for example, we only investigated the effect and mechanism of miR-455-5p on 5-Fu sensitivity in one cell line. Additionally, the role and mechanism of miR-455-5p in 5-Fu sensitivity in CRC cells need to be further verified in 5-Fu-resistant CRC cell line and *in vivo*.
